# Design of a Cooperative Lane Change Protocol for a Connected and Automated Vehicle Based on an Estimation of the Communication Delay

**DOI:** 10.3390/s18103499

**Published:** 2018-10-17

**Authors:** Hongil An, Jae-il Jung

**Affiliations:** Department of Electronics and Computer Engineering, Hanyang University, Seoul 133-791, Korea; aviate@hanyang.ac.kr

**Keywords:** connected and automated vehicle (CAV), lane change, V2V communication sensor, collision detection, path planning

## Abstract

Connected and automated vehicles (CAVs) have recently attracted a great deal of attention. Various studies have been conducted to improve vehicle and traffic safety through vehicle to vehicle (V2V) communication. In the field of CAVs, lane change research is considered a very challenging subject. This paper presents a cooperative lane change protocol, considering the impact of V2V communication delay. When creating a path for a lane change in the local path planning module, V2V communication delay occurs. Each vehicle was represented, in our study, by an oriented bounding box (OBB) to determine the risk of collision. We set up a highway driving simulation environment and verified the improved protocol by implementing a longitudinal and lateral controller.

## 1. Introduction

Autonomous driving technology is attracting substantial global attention from academia and industry. Recently, connected and automated vehicles (CAVs)—which combine vehicle to vehicle (V2V) communication with autonomous vehicles (AV)—have emerged to improve safety and traffic flow efficiency. AVs function, based on self-awareness and self-decision. Since AVs recognize the surrounding environment by utilizing sensors in the vehicle, they are limited by the measurement distance of their sensors. By adding V2V communication, it is possible for an AV to recognize a vehicle at a greater distance and to collaborate with nearby vehicles. Longitudinal control involves the vehicle’s throttle and brake. Lateral control involves the steering of the vehicle [1]. Compared to longitudinal control research, lateral control research has received little attention. Lateral control research has remained relatively unexplored, because it is challenging and requires the exploration of large solution spaces to achieve optimal safety and mobility, while also considering many environmental factors [2].

If V2V communication is combined with AVs, this could provide many advantages to lateral control. First, a major challenge associated with many sensor technologies is that they have a limited sensing range [3]. While an AV equipped with sensors, such as radar and cameras, recognizes the environment within its line-of-sight, a CAV can communicate with remote CAVs outside of its line-of-sight. Second, a CAV can rapidly obtain accurate information on nearby vehicles. When an AV tries to change lanes, nearby AVs in the next lane do not know the intention of the lane change and can only estimate the movement of the AV. However, a CAV equipped with a V2V communication device is able to inform its neighbors of its planned trajectory and arrival time, when undertaking a lane change [4].

Many studies have been undertaken in relation to lane change algorithms. One way to improve the safety of a lane change is to predict the intention and trajectory of nearby vehicles. Schlechtriemen et al. suggested a detection approach, in which an ego vehicle, equipped with a front facing stereo camera and several radar sensors, measures environmental data and recognizes the lane change event [5]. There are also methods to predict lane changes, using past vehicle movements [6,7] and hidden Markov models (HMMs) [8]. However, since these methods are estimation-based, it is difficult to determine the exact intention of the lane changing vehicle from them. In CAVs, V2V communication can be used to convey information related to the vehicle’s intentions and additional data.

Lane change trajectory techniques are classified into two categories [9]: The static and the dynamic planning methods. The dynamic planning method is more precise than the static planning one, due to the fast movement of vehicles. However, since our system focused on V2V communication delay, we applied a simplified static planning method.

There are many collision detection methods [10]. One of these methods—the time-based approach, known as time-to-collision (TTC)—has been successfully tested on the road and used for commercial purposes [11]. However, this measurement does not incorporate the width and length of a vehicle. Thus, we adopted the separating axis theorem (SAT) for collision detection [12].

Some studies have used V2V communication to better optimize lane changes. Dang et al. suggested a method that would show a warning, if vehicles were too close, after analyzing the safety distance between the ego vehicle and rear vehicle in the target lane [13]. Hu proposed a change lanes protocol that accounted for the trade-off between safety and efficiency [14]. However, these methods did not account for the dynamic process of lane changing itself. The contributions of our paper are as follows. First, there are few V2V communication protocols, regarding cooperative lane changes. Previous studies on V2V communication have focused on the sharing of basic vehicle status data, such as basic safety messages (BSMs) or cooperative awareness messages (CAMs) [15]. Second, most studies have not dealt with V2V communication delay. In this paper, we propose a protocol and path-generation module that considers V2V communication delay. We also propose an architecture that integrates V2V communication with the controller, for optimized lane changes.

## 2. Lane Change System for Connected and Automated Vehicles

### 2.1. Overview of Architecture for Lane Changes in CAVs

**Figure 1:**
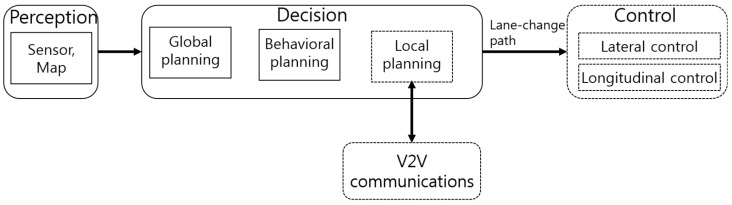
Architecture for a connected and autonomous vehicle (CAV).

The architecture of an autonomous driving system consists of three layers [16]: The perception, decision making, and control layers. In the perception layer, sensors (Global Positioning System (GPS), Light Detection and Ranging (LiDAR), camera, etc.) recognize the surrounding environment and determine the AV’s position on a map. To exchange information with nearby AVs, an AV must be equipped with a V2V communication device. We propose a connected and autonomous, vehicle architecture for lane changes, as shown in Figure 1. In this, we have made various assumptions. First, we assumed that the position of the CAVs in the perception layer was correct. Second, we assumed that the source–destination path was generated in global planning, and that the lane change was determined in behavioral planning. Third, the CAVs did not accelerate during the lane change itself, for safety reasons. In our work, our scope was limited to local planning, V2V communication, and control.

### 2.2. Vehicle Representations

**Figure 2:**
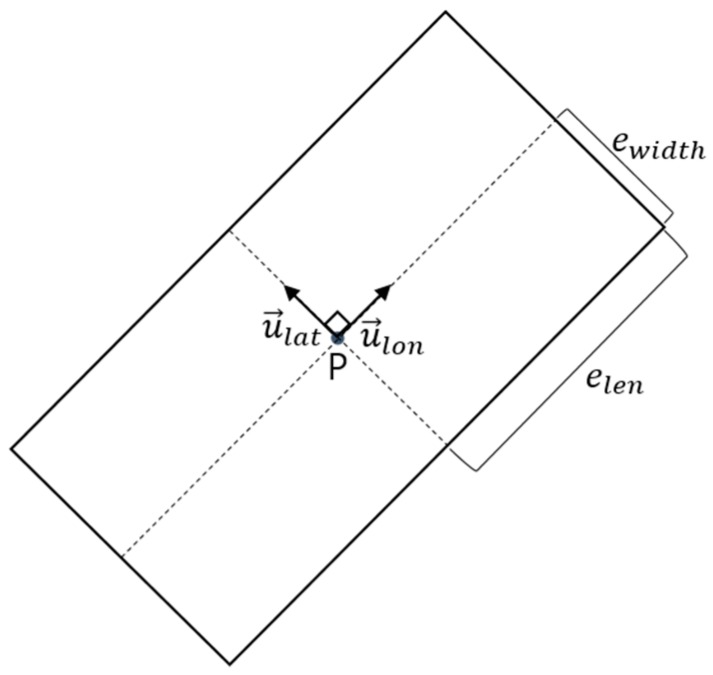
Vehicle representation through an oriented bounding box (OBB) model.

Each vehicle was represented by an oriented bounding box (OBB), as shown in Figure 2. Other studies represented vehicles expressed as circular objects [17]; however, this expression did not properly account for vehicle width and length. In addition, circular objects did not include the heading information of the vehicles. These limitations decreased the accuracy of the prediction in determining whether a collision between vehicles would occur. To improve these weaknesses, we adopted the OBB model.

To express a vehicle as an OBB, the center of the vehicle was represented by a center point P(*t*), at time *t*. A lane change trajectory would then be expressed by the set of points P(*t*):

(1) $$P\left(t\right)=\left[\begin{array}{c} x_{p}\left(t\right)\\ y_{p}\left(t\right) \end{array}\right].$$

The heading of the vehicle was expressed by U. Generally, the heading of a vehicle is defined as an angle. However, we represented the angle with a two-dimensional, unit vector U. Each component of U was perpendicular to the other:

(2) $$U=\left[\begin{array}{cc} {\vec{u}}_{lon} & {\vec{u}}_{lat} \end{array}\right]\;,$$

(3) $${\vec{u}}_{lon}=\left[\begin{array}{c} x_{1}\left(t\right)\\ y_{1}\left(t\right) \end{array}\right],\;{\vec{u}}_{lat}=\left[\begin{array}{c} x_{2}\left(t\right)\\ y_{2}\left(t\right) \end{array}\right].$$

To detect a collision between two OBBs, their respective vehicle lengths and widths were needed. The variable $e_{len}$ is half the vehicle length and $e_{width}$ is half the vehicle width.

### 2.3. Estimation of Communication Delay

In a V2V communication environment, AVs periodically transmit their status information (position, speed, acceleration, etc.). This information is contained in the basic safety message (BSM) format, which complies with the Society of Automotive Engineers (SAE) J2735 standard. In our system, the BSM was utilized to estimate communication delay. The AV created a neighbor table that maintained the latest BSMs of nearby AVs. The end-to-end communication delay of each vehicle could be estimated, through the BSM, in the neighbor table. In our system, communication delays for each vehicle were calculated as a weighted average $t_{w,i}$ and stored in a table. To calculate the V2V communication delays, we modified the formula that estimated the round trip time (RTT) [18]:

(4) $$updated_{t_{w,i}}\leftarrow \left(1-\alpha \right)\times t_{w,i}+\alpha \times t_{r,i}\;,$$

where *i* is the identifier of the CAV; $t_{w,i}$ is the weighted average communication delay of CAV *i*; $t_{r,i}$ is the end-to-end delay of the BSM received from CAV *i*; and α is a constant between 0 and 1. The larger the α, the more $t_{r,i}$ was reflected in the weighted average communication delay $t_{w,i}$. $t_{w,i}$ was recursively calculated for memory space efficiency.

The deviations $dev_{w,i}$ were also calculated to deal with the variability of communication delay:

(5) $$updated\_dev_{t_{w,i}}\leftarrow \left(1-\beta \right)\times {dev}_{w,i}+\beta \times |t_{r,i}-t_{w,i}|.$$

Here, $\left|t_{r,i}-t_{w,i}\right|$ was the difference between the delay of the newly-received BSM and the weighted average communication delay. Essentially, this was a measurement of the fluctuation of the communication delay. The value $dev_{w,i}$ was utilized to compensate for the estimated $t_{w,i}$. The parameter β was similar to α: The larger β, the more the recent fluctuation was reflected in $dev_{w,i}$.

### 2.4. Local Planning for Lane Changes

After deciding to make a lane change in behavioral planning, local planning creates a lane change trajectory, based on the CAV status information. In our local planning, the lane change trajectory was divided into three sections. The first was the preparation section. It was assumed that most lane change studies, using V2V communication, ignored communication delay. However, in real-time automotive systems, V2V communication delays could adversely affect safety. Therefore, when generating the lane change trajectory, CAVs should consider redundant trajectories in dealing with communication delays, processing delays, and other delays. To maintain the same point of view between nodes in communication, three-way handshaking should be performed, as a transmission control protocol (TCP) connection opens [18]. We estimated these delays, and they were included in the lane change trajectory. The preparation delay was defined as the sum of the communication delay, processing delay, and variance:

(6) $$t_{prepare}=3\times \underset{i}{max}t_{w,i}+3\times dev_{w,argmaxt_{w,i}}+t_{processing}.$$

Here, $t_{prepare}$ was the total time that the host CAV—the one trying to change lanes—took, to transmit the expected lane change path, wait for response packets from nearby CAVs, and share the results. In part two of the BSM, there is a path prediction data frame which allows vehicles to share their predicted path trajectory, by estimating the future vehicle path of travel [19]. This data frame was not suitable for representing vehicles as OBBs, nor for detecting collisions in our system. Thus, we defined a new type of message, the definition of which will be discussed in Section 2.5. The quantity $\underset{i}{max}t_{w,i}$ was the largest weighted average delay of the CAVs in the neighbor table, and $argmaxt_{w,i}$ was the ID of the CAV which had the maximum weighted average delay. The value $dev_{w,argmaxt_{w,i}}$ was the standard deviation of the CAV which had the maximum weighted average delay. The value $t_{processing}$ was the time that a nearby CAV took to check whether there would be a collision between expected trajectories and to reply to the host CAV. During the preparation section, we proposed a path with constant velocity linear motion which lasted for $t_{prepare}$. This dealt with the packet delay that was to be exchanged. The next segment of the lane change was the acceleration section. This section dealt with the acceleration required for a collision-free lane change trajectory. Our system was based on constant-velocity motion for stability, requiring acceleration for a lane change in a dynamic driving environment. Acceleration was considered only in this section.

The third and final part was the lane change section. This section defined the actual lane change path. We made use of a ramp sinusoidal function to describe the ideal lane change path [20]:

(7) $$y_{p}\left(t\right)=\frac{y_{e}}{x_{e}}V_{0}t-\frac{a_{y}x_{e}^{2}}{4\pi V_{0}^{2}}\sin\frac{2\pi V_{0}t}{x_{e}}\;(0<t<t_{end}),$$

where $x_{p}$(*t*) = $V_{0}t$ is the linear displacement in the direction of travel, $y_{p}$(*t*) is the lateral displacement (usually equivalent to the lane width), $x_{e}$ is the longitudinal distance for the lane change, $y_{e}$ is the end of the lateral displacement (equivalent to the target lane), $t_{end}$ is the time at which the vehicle arrives in the center of the target lane ($x_{e}$ is equivalent to $V_{0}$ times $t_{end}$), $a_{y}$ is the lateral acceleration, and $V_{0}$ is the current velocity of the CAV. A position in the future lane change trajectory of the vehicle was derived from $x_{p}$(*t)* and $y_{p}$(*t*).

In Reference [20], there was a list of choices for lateral acceleration $a_{y}$. We chose the normal (’feels safe’) lateral acceleration:

(8) $$a_{y}\left(t\right)=\left(0.025V-\left(\frac{V_{0}}{44}\right)\right)g.$$

Further, Limpert proposed a total longitudinal distance $x_{e}$:

(9) $$x_{e}=C_{x}V_{0}\sqrt{\frac{y_{e}}{a_{y}}}.$$

Limpert [21] cited experimental results that yielded a distribution of values for the constant coefficient $C_{x}$, with the mode at 2.67. Based solely on algebraic manipulation, $C_{x}$ was approximately equal to 2.51.

**Figure 3:**
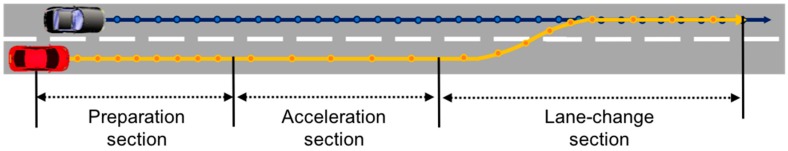
Construction of the expected lane change trajectory.

Figure 3 shows the expected lane change trajectory, generated by our local planning approach.

### 2.5. Cooperative Lane Change Protocol

A cooperative lane change protocol was the most important part of our system. This protocol was based on dedicated short-range communication (DSRC) radios, using the IEEE 802.11p/WAVE standard [22]. All CAVs were equipped with DSRC radios and sensors, which allowed them to perceive and communicate their surroundings to other vehicles.

**Figure 4:**
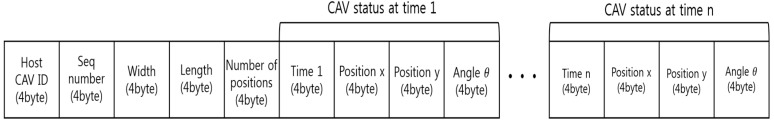
Definition of the lane change request message.

For a cooperative lane change, a host CAV wanting to change lanes received a lane change trajectory from a local planning module. The host CAV converted the lane change trajectory into a set of discrete positions, through a sampling process, and added it to the set of discrete positions from the preparation and acceleration sections. The sampling process is described in Section 2.6. Then, the host CAV created a lane change request message, which had a host CAV ID, a sequence number of the lane change request, the width and length of the CAV, and the number of discrete positions. Furthermore, the request message contained a set of times, discrete positions, and angles (*t*, *x*, *y*, θ) of the combined expected trajectory. The structure of the message is outlined in Figure 4. The host CAV then broadcasted the lane change request message to nearby CAVs. Nearby CAVs compared their expected trajectory with the set of the host CAV in the lane change request message, to determine the risk of collision. CAVs return the result of their collision assessments, and the host CAV attempted a lane change, only if all the nearby CAVs in the preparation delay did not broadcast a risk of collision. The following algorithms show the cooperative lane-change protocol for the host and nearby CAVs. The protocol needed to have been completed within the preparation period $t_{prepare}$. Algorithm 1 shows the procedure in the host CAV, wanting to change to the next lane. Algorithm 2 shows the procedure in each nearby CAV, after receiving a lane change request message from the host CAV.

**Algorithm 1.** Lane change protocol in the host CAV**for**$V_{target}$ = $V_{current}$
**to**
$V_{max}$
**do**  Generate the preparation path at the constant velocity $V_{current}$ for $t_{prepare}$  Generate the acceleration path at the uniform acceleration $a_{max}$ from $V_{current}$ to $V_{target}$  Generate the lane change path drawn with $y_{p}$(*t*) function  Combine the preparation path, the acceleration path, and the lane change path  Convert the combined continuous path into discrete positions at intervals of 0.1 s  Make a lane change request message  Send the lane change request message to nearby CAVs  **repeat**    Receive the response packets from nearby CAVs    Until Receive Negative Acknowledgment or OK from all nearby CAVs    **if**
$t_{current}$ > $t_{prepare}$
**then**      Continue **for loop**  **if** Receive Negative Acknowledgment **then**    Continue **for loop**  **else**    Pass the path to the controller    Exit lane change protocol  **end if**  Send the last Acknowledgment packet for 3-way **end for**

**Algorithm 2.** Lane change protocol in the nearby CAVReceive path from the host CAV Generate its expected path at the constant velocity $V_{current}$ Check for collision between path of the host CAV and path of its expected path **if** Detect collision **then**  Send Negative Acknowledgment response **else**  Send OK response **end if** Wait for the last Acknowledgment packet

### 2.6. Sampling Lane Change Trajectory

A sampling process was required to deliver a continuous trajectory to the nearby CAVs in a packet. It was not possible for a packet to include the continuous trajectory. Therefore, we extracted discrete points from the continuous path, at specific time intervals. This process was called sampling. After the sampling process, a CAV could send their expected trajectory to other CAVs. 

### 2.7. Collision Detection between Expected Paths

CAVs receiving the expected lane change path likewise generated and sampled the expected path. The risk of collision was analyzed, using two sampled trajectories. In our system, the sampled point was the center of the CAV, which was represented by the OBB. The collision detection problem was transformed into a collision detection between two OBBs. For this purpose, the separating axis theorem (SAT) was applied. SAT followed from the separating hyperplane theorem. It stated that, given two convex sets, A and B, either the two sets were intersecting or there existed a separating hyperplane P, such that A was on one side of P and B was on the other [23]. In our system, the OBBs each belonged to a convex set. Because the two OBBs were represented in two dimensions, if we found a one-dimensional line which separated two OBBs, that line was a hyperplane P. As a result, if there was a one-dimensional line separating the two OBBs, corresponding to the two vehicles, there was no collision.

A test for OBB–OBB intersection could be implemented in terms of what is known as the separating axis test [23]. It states that two OBBs are separate, if—with respect to two axes per OBB—the sum of their projected radii is less than the distance between the projection of their OBBs center points. For an OBB–OBB intersection test in two dimensions, a total of four separating axis tests should be performed.

### 2.8. Controller

When the host CAV received OK packets from all nearby CAVs, it passed its expected trajectory to the controller. The role of the controller was to track the position of the CAV at a given time. The continuous lane change trajectory was sampled at discrete points, at corresponding discrete time intervals. In the controller, the difference between the current position and the target position was regarded as an error at that specific moment, and the vehicle was controlled in such a direction as to minimize the difference. The difficulty of a lane change control maneuver was that both the longitudinal and lateral aspects needed to have been considered. In a typical system, which gives a trajectory to follow, without being given a specific time, we could simply consider the steering controller. However, our system considered both the longitudinal and lateral aspects, as we had to reach a certain point at a certain time.

For longitudinal control, proportional–integral–derivative (PID) control was applied to our system. We calculated the expected speed from the current position, the expected position, and the sampling period. The PID error was derived from the expected speed and current speed. For the lateral control, the steering wheel was manipulated by applying pure pursuit control [24]. The pure pursuit control algorithm determined the steering angle, by using the look-ahead distance to follow the continuous path. However, as our system followed the discrete path, we could not calculate the angle using the look-ahead distance. We replaced the look-ahead distance with the look-ahead point, which was one of the expected points in the sampled trajectories.

## 3. Evaluation

### 3.1. Setup

**Figure 5:**
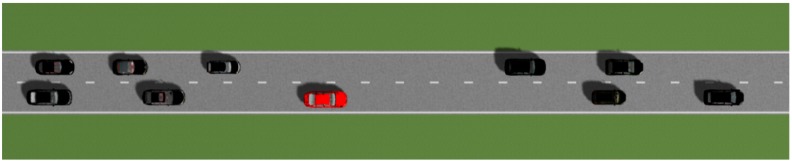
Screenshot of the road scenario.

Prescan was used for the CAV experimental environment. In the field of vehicular ad hoc network (VANET) research, the experimental environment was mainly set up by linking a network simulator to a traffic simulator. However, since the main purpose of the traffic simulator was macroscopic analysis, it was not suitable for microscopic analysis, such as analyzing lane change events (i.e., when a vehicle changes a lane in the traffic simulator, it moves as if teleporting from one lane to the other). In our paper, we adopted Prescan, because the progress of the lane change in the simulator could be controlled and the V2V communication function utilized. Each vehicle was equipped with a V2V communication device that complied with IEEE 802.11p/WAVE standard. Prescan was not as rich as the network simulator, in terms of communication simulation. Due to the limitations of the communication functions, the V2V communication delay was assumed to follow a normal distribution. The road driving scenario consisted of 10 CAVs on a highway road type. Figure 5 shows a screenshot corresponding to the road driving scenario. The given scenario was one in which a red CAV tried to change lanes at a particular time. The parameters related to the simulation are defined in Table 1.

### 3.2. Simulation Results

**Figure 6:**
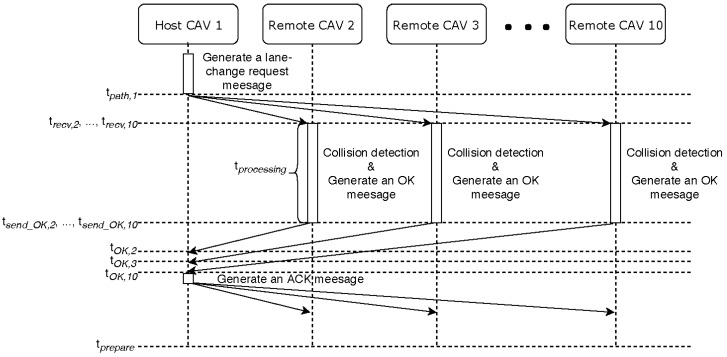
Time sequence diagram of the lane change protocol in the preparation section.

**Figure 7:**
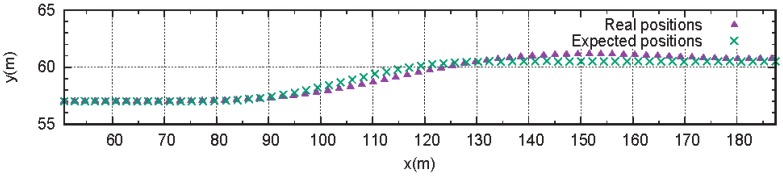
Real positions and expected positions of the host CAV in the lane change.

We demonstrated the effectiveness of the proposed system through simulations on highways with two lanes. The red CAV, shown in Figure 5, accomplished the left lane change, after a total of three attempts, at a target speed of 22 m/s. The first two failures were due to an expected collision between the planned paths. In these instances, it was predicted that the red CAV would collide, while moving to the left lane. On the last attempt, all CAVs communicated lane change packets through V2V communication, during the preparation section. Figure 6 and Table 2 show the working process of the lane change protocol, through a time sequence diagram. $t_{send,1}$ was the time at which the red CAV with ID 1 sent the sampled path. $t_{recv,i}$ was the time at which CAV *i* received the sampled path from the red CAV. $t_{send\_OK,i}$ was the time that the nearby CAVs sent an OK packet, if there was no collision between the future trajectories. $t_{OK,i}$ was the time that the red CAV received the OK packet from CAV *i*. $t_{ACK,1}$ was the time that the red CAV sent the acknowledgement (ACK) packet, as all CAVs consented to the lane change suggestion. Figure 7 shows the expected position and actual position of the red CAV. Before attempting a lane change, the CAV created a predicted path, taking into account the V2V communication delay. Even in a communication environment in which there is a V2V communication delay, the CAV driven by the PID and pure pursuit controllers, proposed in our system, did a very good job of following the expected path.

## 4. Conclusions

This paper presented the architecture and protocol of lane changes for CAVs. In addition, we proposed a system that could accurately convey the lane change intention through V2V communication, instead of estimating the it through sensors and statistical models. The most important contribution of this paper was the consideration of the V2V communication delay, when a CAV creates a lane change trajectory, as many studies have assumed no communication delay [25,26,27]. A lane change protocol for CAVs was also proposed.

Some challenging scenarios were designed to be applicable in the field. We suggest future research projects to further improve the proposed system’s performance. First, the packet loss situation should be considered in V2V communication. To cope with packet loss, complex logic, such as retransmission, must be added. Second, when creating a lane change path, vehicle dynamics must be considered. If the dynamic model is applied to the expected path, the error between the expected path and the actual path could be reduced.

## Figures and Tables

**Table 1:** Parameters in the lane change simulation.

Parameter	Value
Simulation time	30 s
Number of CAVs	10
Length of road	300 m
Number of lanes	2
Width of lanes $y_{e}$	3.5
Initial velocity	20 m/s
$a_{max}$	0.3 g m/$s^{2}$
$a_{y}$	2.62 m/$s^{2}$
Width of CAVs	2.04 m
Length of CAVs	5.21 m
Mean of normal distribution	50
Standard deviation	15
Sampling rate	10
$K_{p}$	20
$K_{i}$	20
$K_{d}$	10
Look up points in pure pursuit	8
$C_{x}$	2.51
$t_{processing}$	100 m/s
α	0.125
β	0.25

**Table 2:** Symbols in the preparation section.

Symbol	Value
$t_{path,1}$	0.966 s
$t_{recv,2},\;\ldots ,t_{recv,10}$	0.998 s
$t_{OK,2}$	1.144 s
$t_{OK,3}$	1.146 s
$t_{OK,4}$	1.146 s
$t_{OK,5}$	1.144 s
$t_{OK,6}$	1.148 s
$t_{OK,7}$	1.147 s
$t_{OK,8}$	1.146 s
$t_{OK,9}$	1.144 s
$t_{OK,10}$	1.148 s
$t_{ACK,1}$	1.149 s
